# Plasma proteomic profiling of AD, PD and FTD in a large sample of individuals from the global neurodegeneration proteomics consortium (GNPC)

**DOI:** 10.1002/alz70856_100576

**Published:** 2025-12-25

**Authors:** Muhammad Ali, Yike Chen, Ying Xu, Gyujin Heo, Jigyasha Timsina, Menghan Liu, Yun Ju Ju Sung, Alexa Pichet Binette, Betty M. Tijms, Laura M Winchester, Carlos Cruchaga, Global neurodegeneration proteomics consortium (GNPC)

**Affiliations:** ^1^ Department of Psychiatry, Washington University School of Medicine, St. Louis, MO, USA; ^2^ NeuroGenomics and Informatics Center, Washington University School of Medicine, St. Louis, MO, USA; ^3^ Clinical Memory Research Unit, Department of Clinical Sciences Malmö, Faculty of Medicine, Lund University, Lund, Sweden; ^4^ Alzheimer Center, Department of Neurology, Amsterdam university medical center, Amsterdam, Netherlands; ^5^ Department of Psychiatry, University of Oxford, Oxford, United Kingdom; ^6^ Hope Center for Neurological Disorders, Washington University in St. Louis, St. Louis, MO, USA; ^7^ The Charles F. and Joanne Knight Alzheimer Disease Research Center, St Louis, MO, USA; ^8^ Washington University School of Medicine, St. Louis, MO, USA

## Abstract

**Background:**

The Global Neurodegeneration Proteomics Consortium (GNPC) aims to advance understanding of neurodegenerative diseases (ND) through comprehensive collaborative proteomic analysis. Here, we present the first analyses from the cross‐sectional profiling workstream, in which we studied disease specific plasma proteomic profiles across *n* = 9,963 individuals.

**Method:**

The cross‐sectional profiling workstream utilizes a diverse set of ND cohorts, including samples from Alzheimer's disease (AD), Parkinson's disease (PD), and Frontotemporal dementia (FTD). Protein expression levels for up to 7,288 analytes were measured using the SomaScan platform. Separate linear regression models were employed to evaluate differences in plasma protein levels between each clinically defined ND (AD, PD, FTD) and the control group, adjusting for covariates such as age, sex, contributor site, and the first two proteomic principal components. To account for multiple testing, *p*‐values were adjusted using the false discovery rate.

**Result:**

The largest plasma proteomic dataset (AD = 1,936, PD = 2,186, FTD = 65, Controls = 5,776) was leveraged to identify significantly altered proteins across different ND. Compared to controls, AD exhibited the most extensive proteomic alterations, with over 1,000 analytes (>15%) showing significant changes, including elevated levels of SPC25 (Estimate=0.23, *p* = 1.1×10^‐27^) and SMOC1 (Estimate=0.54, *p* = 6.4×10^‐82^). In PD, more than 150 analytes showed altered levels, featuring increased levels of PCBP3 (Estimate=0.13, *p* = 1.1×10^‐04^) and SUMF1 (Estimate=0.52, *p* = 6.9×10^‐17^). FTD had over 50 analytes with significant changes, including decreased levels of NPTXR (Estimate=‐0.59, *p* = 6.5×10^‐09^) and OMG (Estimate=‐0.74, *p* = 5.9×10^‐12^). While each disease displayed a distinct proteomic signature, AD and PD shared the highest number of overlapping differentially abundant proteins compared to controls.

**Conclusion:**

The GNPC's cross‐sectional workstream represents a significant step forward in ND research. By comparing proteomic data across diseases, the workstream enhances our understanding of the molecular underpinnings of neurodegeneration and opens opportunities for more precise and accurate disease subtyping.

